# Cell-cycle arrest biomarkers in urine to predict acute kidney injury in septic and non-septic critically ill patients

**DOI:** 10.1186/s13613-017-0317-y

**Published:** 2017-09-07

**Authors:** M. Cuartero, J. Ballús, J. Sabater, X. Pérez, N. Nin, J. Ordonez-Llanos, A. J. Betbesé

**Affiliations:** 1 Intensive Care Department and Institut d’Investigacions Biomèdiques, Hospital de la Santa Creu i Sant Pau, Universitat Autònoma de Barcelona, Sant Quintí 89, 08041 Barcelona, Spain; 2 Intensive Care Department, Hospital Universitari Bellvitge, Hospitalet de Llobregat, Universitat de Barcelona, Feixa Llarga, 08907 L’Hospitalet de Llobregat, Barcelona, Spain; 3 Intensive Care Department, Hospital Universitario de Getafe, Km 12500 Madrid - Toledo Road, 28905 Getafe, Madrid, Spain; 4 Biochemistry Department and Institut d’Investigacions Biomèdiques, Hospital de la Santa Creu i Sant Pau, Universitat Autònoma de Barcelona, Sant Quintí 89, 08041 Barcelona, Spain

**Keywords:** Acute kidney injury, Biomarkers, Tissue inhibitor of metalloproteinases-2, Insulin-like growth factor-binding protein 7, ICU patients, Sepsis

## Abstract

**Purpose:**

To analyse the usefulness of the composite index of the tissue inhibitor of metalloproteinases-2 (TIMP-2) and insulin-like growth factor-binding protein 7 (IGFBP7) as urinary biomarkers for the early prediction of AKI in septic and non-septic patients.

**Methods:**

This is a prospective, observational study including patients admitted to ICU from acute care departments and hospital length of stay <48 h. The main exclusion criteria were pre-existing eGFR <30 mL/min/1.73 m^2^ and hospitalisation 2 months prior to current admission. The [TIMP-2]·[IGFBP7] index was analysed twice, within the first 12 h of ICU admission.

**Results:**

The sample included 98 patients. AKI incidence during ICU stay was 50%. Sepsis was diagnosed in 40.8%. Baseline renal variables were comparable between subgroups except for a higher baseline eGFR in non-septic patients. Patients were stratified based on the presence of AKI and their highest level of [TIMP-2]·[IGFBP7] within the first 12 h of stay. [TIMP-2]·[IGFBP7] index values were dependent on the incidence of AKI but not of sepsis. [TIMP-2]·[IGFBP7] values were significantly related to AKI severity according to AKIN criteria (*p* < 0.0001). The AUROC curve to predict AKI of the worst [TIMP-2]·[IGFBP7] index value was 0.798 (sensitivity 73.5%, specificity 71.4%, *p* < 0.0001). Index values below 0.8 ruled out any need for renal replacement (NPV 100%), whereas an index >0.8 predicted a rate of AKI of 71% and AKIN ≥ 2 of 62.9%.

**Conclusions:**

In our study, urinary [TIMP-2]·[IGFBP7] was an early predictor of AKI in ICU patients regardless of sepsis. Besides, index values <0.8(ng/mL)^2^/1000 ruled out the need for renal replacement.

**Electronic supplementary material:**

The online version of this article (doi:10.1186/s13613-017-0317-y) contains supplementary material, which is available to authorized users.

## Background

 Acute kidney injury (AKI) is a frequent complication in ICU patients [1]. AKI definitions based on serum creatinine (sCr) have shown a large variation in AKI incidence and associated outcomes [2]. However, it has been observed that AKI requiring renal replacement therapy (RRT) is an independent factor of poor outcome [3, 4], with an associated mortality rate of 50–60% [2, 3].

Sepsis is a relevant contributing factor to AKI development. AKI is present in >30% of those with sepsis [5, 6] and in >50% of those with septic shock [7, 8]. Pathogens producing sepsis and their toxins affect the whole body as well as specific organs. Damaging molecules can reach the proximal renal tubular cells in high concentrations and may trigger kidney injury followed by inflammation and oxidative stress and, finally, cell damage [9].

Diagnosis of AKI has changed in the last decade with the advent of RIFLE [10], AKIN [11] and KDIGO [12] classifications. These tools are based on sCr and diuresis. However, sCr is a late and non-specific AKI biomarker [13, 14]. Biomarkers that can rapidly and specifically recognise AKI are therefore needed. A few recent studies have shown that tissue inhibitor of metalloproteinases-2 (TIMP-2) and insulin-like growth factor-binding protein 7 (IGFBP7) are specific biomarkers of structural renal damage in critically ill patients [15, 16]. Renal tubular cells enter in G_1_ cell arrest to block the effects of molecules contributing to cell-cycle promotion, such as cyclins. This mechanism prevents the extension of cell damage. TIMP-2 and IGFBP7 are protective molecules involved in G_1_ cell-cycle arrest that moderate apoptotic, angiogenic [17], inflammatory [18, 19] and ischaemic processes [20]. Since renal cell arrest usually occurs 24–48 h before sCr rises due to a significant fall in the glomerular filtration rate, TIMP-2 and IGFBP7 are thought to be earlier AKI biomarkers than sCr.

TIMP-2 and IGFBP7 are detectable in urine. Previous studies in unselected ICU populations have shown that when analysed together as the index [TIMP-2]·[IGFBP7], they perform better than sCr, urine and plasma NGAL, plasma cystatin-C and KIM-1 for early detection of AKI and improved risk stratification for renal and general outcomes [15, 16]. The aim of this study was to assess whether values of the TIMP-2 and IGFBP7 index were early predictors of AKI in a selected ICU population free of the most frequent AKI risk factors. To date, the influence of sepsis on TIMP-2 and IGFBP7 expression in critically ill patients is not well defined. As a secondary objective, we thus also evaluated the potential usefulness of [TIMP-2]·[IGFBP7] index in septic ICU patients.

## Methods

The study protocol was approved by the institutional review board at each participating centre. We obtained informed consent from patients or their guardians. The study prospectively included patients over 18 years of age with an expected ICU stay of at least 48 h. We included patients admitted to ICU either from the emergency department or after undergoing acute surgery. Exclusion criteria were pregnancy, established anuric AKI, pre-existing chronic kidney disease (CKD) with estimated glomerular filtration rate (eGFR) <30 mL/min/1.73 m^2^ and current hospital length of stay >48 h. Medical management was left to the discretion of the attending physicians. All healthcare providers involved were blinded to the biomarkers results.

### Sampling and measurement of the [TIMP-2]·[IGFBP7] index

Urine samples for biomarker analysis were collected twice: at ICU admission and up to 12 h later simultaneously with the morning blood work. Urine samples were centrifuged, and supernatants were frozen at ≤−70 °C and stored until analysed. Before analysis in a central laboratory at Hospital de la Santa Creu i Sant Pau, aliquots were thawed at room temperature and centrifuged at 3000 rpm for 15 min. A previous study in the central laboratory showed there were no significant differences in the [TIMP-2]·[IGFBP7] index between fresh and frozen urine samples (data not shown). The [TIMP-2]·[IGFBP7] index was measured by a sandwich fluorescent quantitative immunoassay adapted to a portable device (Nephrocheck^®^, Astute Medical). All samples were analysed in the same batch to avoid between-batch variability. The portable device provides a [TIMP-2]·[IGFBP7] index in ((ng/mL)^2^/1000) units. According to the manufacturer, an index ≤0.3 suggests a low risk of AKI, values between 0.3 and 2.0 suggest a high risk, and values >2.0 suggest a very high risk [15].

### Data collection

Clinical data included patient demographics and comorbidities. The APACHE II score, the SAPS II and the SOFA score were recorded at ICU admission. Patients were classified following the AKIN classification [11], which includes changes in sCr and urine output. Baseline sCr was taken from patients’ pre-admission records whenever possible and used to estimate eGFR before ICU admission using the Cockcroft–Gault formula. When baseline sCr was not available, AKI was defined only with AKIN urine output criterion. Sepsis and septic shock were defined according to standard criteria [1, 21].

### Statistical analysis

IBM^®^ SPSS^®^ version 21 (IBM corp., Armonk, NY) was used. Variables with Gaussian distribution are reported as mean ± standard deviation and were compared with the Student’s *t* test or one-way analysis of variance. Variables with a non-Gaussian distribution are reported as median and interquartile range (IQR) and were compared with Mann–Whitney’s *U* or Kruskal–Wallis tests. Categorical data are reported as percentage and were compared using Chi-square test or Fisher exact test.

The primary outcome of the study was AKI prediction with the [TIMP-2]·[IGFBP7] index. For statistical analyses, we used the highest value observed during the first 12 h of ICU admission as the worst index. Reporting of results followed the Standards for Reporting Diagnostic Accuracy Studies (STARD) statement. As [TIMP-2]·[IGFBP7] index provides a quantitative result that it is interpreted with two cut-offs, analysis was performed to establish the diagnostic accuracy of the test in comparison with the reference standard, which is the AKIN definition of AKI. Diagnostic values, positive predictive values (PPV) and negative predictive values (NPV) were assessed a priori for AKI statistically relevant variables by the area under the receiver operator characteristic curve (AUROC) and by the odds ratio (OR). Results were presented with a 95% confidence interval and probability (*p*). For selected thresholds of [TIMP-2]·[IGFBP7], sensitivities, specificities, PPV and NPV were reported for the worst [TIMP-2]·[IGFBP7] value within the first 12 h in the ICU. The ROC analysis was used to calculate the best [TIMP-2]·[IGFBP7] cut-off point for AKI and AKIN ≥ 2 prediction. A *p* < 0.05 was considered significant.

## Results

**Fig. 1:**
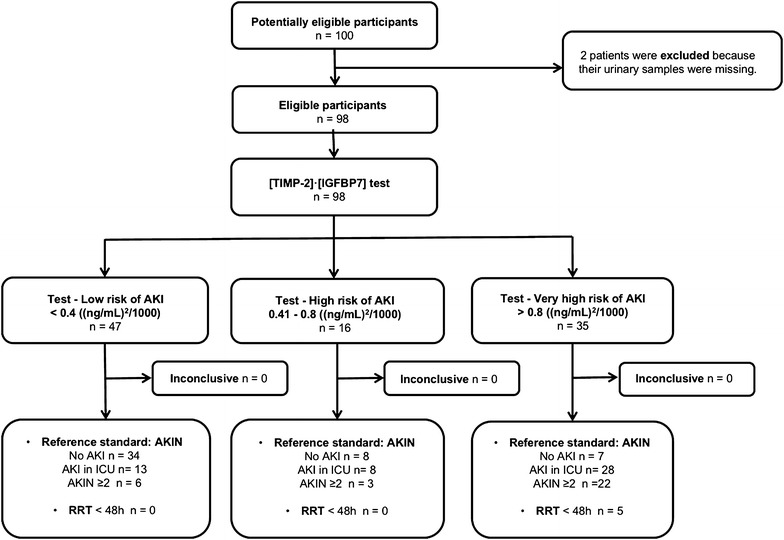
Diagram of [TIMP-2]·[IGFBP7] index to predict AKI, study enrolment and inclusion

We recruited 100 consecutive patients fulfilling the admission criteria in the ICU of two university hospitals, from June 2011 to April 2013. Two patients were excluded from the study because one of their samples was missing (Fig. 1). Table 1 includes the main characteristics and outcomes of the overall study population as well as in subgroups depending on the occurrence of AKI and sepsis at admission. Further epidemiological data are included in Additional file 1: Table S1.

**Table 1:** Main characteristics of the study population in subgroups of AKI/non-AKI and septic/non-septic patients

	Total	Non-AKI	AKI	*p* value	Non-septic	Septic	*p* value
*n*	98	49	49	–	58	40	–
Men	65 (66.3%)	30 (61.2%)	35 (71.4%)	NS	38 (65.5%)	27 (67.5%)	NS
Age (years)	55 ± 17.3	50.4 ± 17.9	59.9 ± 15.5	0.006	50.9 ± 16.6	61.2 ± 16.8	0.003
Renal characteristics							
Baseline eGFR (mL/min) *n* = 86	110.6 ± 53.7	120.5 ± 60.5	100.7 ± 44.6	NS	121.9 ± 58.9	94.8 ± 41.2	0.020
Baseline creatinine (μmol/L) *n* = 86	79 ± 31.5	80.5 ± 32.3	78.1 ± 31.1	NS	79.6 ± 28.9	78.9 ± 35.1	NS
Creatinine upon ICU admission	94.1 ± 42.2	74.6 ± 30.2	113.1 ± 43.9	<0.001	85.0 ± 36.8	106.9 ± 46.5	0.015
AKI at admission	44 (44.9%)	–	44 (89.8%)	–	20 (34.5%)	24 (60%)	0.013
AKIN ≥ 2 upon ICU admission	25 (25.5%)	–	25 (51%)	–	9 (15.5%)	16 (40%)	0.009
Renal replacement <48 h	5 (5.1%)	0 (0%)	5 (10.2%)	0.056	4 (6.9%)	1 (2.5%)	NS
Worst value [TIMP-2]·[IGFBP7] ((ng/mL)^2^/1000)	0.41 (0.20–1.36)	0.24 (0.11–0.48)	1.03 (0.38–3.29)	<0.001	0.36 (0.14–1.08)	0.56 (0.26–2.94)	NS
ICU epidemiological data							
Shock	35 (35.7%)	11 (22.5%)	24 (50%)	0.011	10 (17.2%)	25 (62.5%)	<0.001
Septic shock	19 (19.4%)	6 (12.3%)	13 (26.5%)	NS	–	19 (47.5%)	–
Mechanical ventilation	79 (80.6%)	39 (79.6%)	40 (81.6%)	NS	44 (75.9%)	35 (87.5%)	NS
SAPS II	37.4 ± 18.3	30.9 ± 14.9	43.9 ± 19.2	<0.001	30.9 ± 15.6	46.8 ± 18.1	<0.001
APACHE II	15.7 ± 8.2	13.5 ± 7.7	17.9 ± 8.2	0.007	14.2 ± 8.5	17.9 ± 7.3	0.024
SOFA at ICU admission	7.5 ± 3.7	6.1 ± 3.2	8.9 ± 3.7	<0.001	6.7 ± 3.7	8.7 ± 3.4	0.009
ICU LOS (days)	11.1 ± 14.6	10.0 ± 10.6	12.3 ± 18.8	NS	9.3 ± 11.0	13.9 ± 18.5	NS
Hospital LOS (days)	23.6 ± 25.0	23.4 ± 23.3	23.8 ± 27.0	NS	21.4 ± 21.3	26.9 ± 29.6	NS
ICU mortality	10 (10.2%)	4 (8.2%)	6 (12.3%)	NS	6 (10.3%)	4 (10%)	NS

Values expressed as either % per column, mean ± standard deviation or median and interquartile range. *p* value of statistical significance

*NS* no statistical significance, *AKI* acute kidney injury, *AKIN* acute kidney injury network definition, *APACHE II* acute physiology and chronic health evaluation II, *ICU* intensive care unit, *LOS* length of stay, *SAPS II* Simplified Acute Physiology Score II, *SOFA* sequential organ failure assessment score

At admission, 44 patients presented some grade of AKI, more frequently observed in septic patients; 19 fulfilled criteria for AKIN 1, 20 AKIN 2 and 5 AKIN 3. Throughout ICU admission AKI affected 49 of 98 patients. The incidence of AKIN ≥ 2 increased from 25.5 to 31.6% in those patients who presented AKI. Five of 98 patients required RRT within the first 48 h of admission. 40.8% had sepsis at ICU admission.

When comparing subgroups depending on the occurrence of AKI and sepsis in ICU (Table 1; Additional file 1: Table S1), those patients in AKI or sepsis categories were older and had a higher incidence of shock and higher severity scores. Although the incidence of shock in both subgroups was higher, there was no statistical difference in the incidence of septic shock between the non-AKI and AKI subgroups. Septic patients also showed a lower baseline eGFR. However, baseline sCr and the remaining patients’ characteristics for both subgroups did not differ.

Overall mortality was 10.2% in ICU, and 12.2 and 13.3%, respectively, at 28 and 90 days (Table 1; Additional file 1: Table S1).

### Composite index of [TIMP-2]·[IGFBP7]

[TIMP-2]·[IGFBP7] index values were significantly higher in patients with AKI (1.03, IQR 0.38–3.29) than in those without AKI (0.24, IQR 0.11–0.48) (*p* < 0.001) (Table 1). These differences were unrelated to the presence of sepsis (Table 2). Patients who developed AKI presented higher median index values (1.05, IQR 0.41–2.31 for patients without sepsis; 0.98, IQR 0.36–3.94 for septic patients) than those without AKI (0.21 IQR 0.10–0.40 in non-septic patients and 0.32 IQR 0.15–0.63 for those with sepsis) with *p* < 0.001 between subgroups with and without AKI (no statistical differences were found between septic and non-septic status).

**Table 2:** Worst [TIMP-2]·[IGFBP7] distribution within 12 h of ICU admission depending on AKI and sepsis

	AKI −	*p* value	AKI +
Sepsis −	0.21 (0.10–0.40) (*n* = 35)	⇐ *p* < 0.001 ⇒	1.05 (0.41–2.31) (*n* = 23)
*p* value	⇑ NS ⇓		⇑ NS ⇓
Sepsis +	0.32 (0.15–0.63) (*n* = 14)	⇐ *p* = 0.009 ⇒	0.98 (0.36–3.94) (*n* = 26)

(AKI + vs. AKI −) and (Sepsis + vs. Sepsis −) represent the presence or absence of either AKI during hospital stay or sepsis upon admission, respectively. Values show median and percentiles 25–75. *p* represents the statistical intra-group differences. [TIMP-2]·[IGFBP7] values given in ((ng/mL)^2^/1000)

*AKI* acute kidney injury, *NS* no statistical differences

**Fig. 2:**
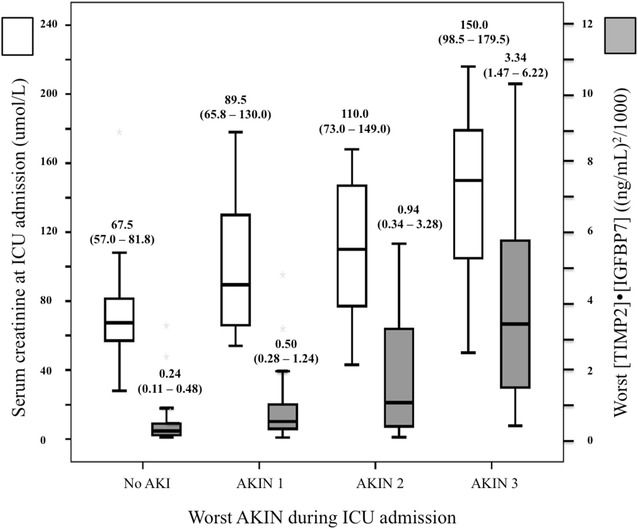
Boxplot comparing the worst [TIMP-2]·[IGFBP7] index and sCr upon admittance with the worst AKIN. *Boxplots* indicate the median, 25th and 75th percentiles. Whiskers indicate the 5th and 95th percentiles. Statistical significance (*p*) comparing each biomarker index with AKIN and RIFLE categories. *p* was determined by Kruskal–Wallis test, not correlation. We found statistical differences in the worst [TIMP-2]·[IGFBP7] index concentrations when comparing the subgroup without AKI with any degree of AKI defined by AKIN. Between non-AKI and AKIN 1, *p* was 0.014 and *p* < 0.001 when comparing with AKIN 2 and AKIN 3. In between AKIN 1 and AKIN 3, *p* = 0.004, whereas in between AKIN 2 and AKIN 3 *p* = 0.039. When comparing sCr levels with AKIN, we found differences between the subgroup without AKI with AKIN 1 (*p* = 0.008), AKIN 2 and 3 (for both *p* < 0.001). In between AKIN 1 and 3, *p* was 0.048 and *p* = 0.033 in AKIN 2 versus AKIN 3

The index values were significantly related to AKI presence and severity according to AKIN criteria (Fig. 2). In contrast, index values did not appear to be influenced by sepsis, either in AKI or non-AKI patients (Table 2).

[TIMP-2]·[IGFBP7] values increased in 44% of patients within 12 h of ICU admission; index values decreased in all other patients. Overall, a repeated determination of [TIMP-2]·[IGFBP7] did not show any differences between non-AKI/AKI and non-septic/septic subgroups (Additional file 2: Figure S1).

We divided the patients into 3 subgroups depending on the cut-offs proposed in previous studies [15] (Table 3). Although baseline sCr showed no statistically significant difference between subgroups, baseline eGFR significantly declined at index values >2.0. AKI occurrence and AKIN ≥ 2 during ICU stay were significantly more frequent in the groups with index >0.31. However, the need for RRT did not differ according to the index values. There were also differences between subgroups in the incidence of shock, SAPS II score and ICU length of stay. Patients with high-risk values also had longer ICU length of stay but not hospital length of stay, both with wide standard deviation. According to the manufacturer’s [TIMP-2]·[IGFBP7] stratification, both subgroups of patients with high and very high-risk values needed RRT. There were no differences between subgroups regarding ICU mortality or mortality at 28 and 90 days.

**Table 3:** Population characteristics depending on different cut-offs of [TIMP-2]·[IGFBP7] index for AKI diagnose

	Worst [TIMP-2]·[IGFBP7] index within the first 12 h in ((ng/mL)^2^/1000)							
	≤0.3	0.31–2	>2	*p* value	≤0.4	0.41–0.8	>0.8	*p* value
*n*	38	40	20	–	47	16	35	–
Men (*n* = 65)	21 (55.3%)	30 (75%)	14 (70%)	NS	29 (61.7%)	12 (75%)	24 (68.6%)	NS
Age (years)	53 ± 18.4	54 ± 16.7	62 ± 15	NS	52 ± 18.5	52 ± 18.4	61 ± 13.7	0.043
Renal characteristics								
Baseline creatinine (μmol/L)	84 ± 31.9	72 ± 28.5	85 ± 34.9	NS	83 ± 30.1	66 ± 33.3	80 ± 32	NS
Baseline eGFR (mL/min) *n* = 86	117 ± 60.1 (*n* = 31)	123 ± 48.1 (*n* = 35)	79 ± 40.9 (*n* = 20)	0.008	112 ± 55.4 (*n* = 39)	128 ± 54.1 (*n* = 12)	103 ± 52.7 (*n* = 35)	NS
AKI while in ICU (*n* = 49)	9 (23.7%)	22 (55%)	18 (90%)	<0.001	13 (27.7%)	8 (50%)	28 (80%)	<0.001
AKIN ≥ 2 while in ICU (*n* = 31)	4 (10.5%)	13 (32.5%)	14 (70%)	<0.001	6 (12.8%)	3 (18.8%)	22 (62.9%)	<0.001
RRT <48 h (*n* = 5)	0 (0%)	3 (7.5%)	2 (10%)	NS	0 (0%)	0 (0%)	5 (14.3%)	0.007
ICU epidemiological data								
Sepsis at admission (*n* = 40)	11 (29%)	18 (45%)	11 (55%)	NS	16 (34%)	8 (50%)	16 (45.7%)	NS
Shock (*n* = 35)	11 (29%)	12 (30%)	12 (60%)	0.039	13 (27.7%)	4 (25%)	18 (51.4%)	0.053
Mechanical ventilation (*n* = 79)	32 (84.2%)	32 (80%)	15 (75%)	NS	39 (83%)	13 (81.3%)	27 (77.1%)	NS
SAPS II	35 ± 15.4	34 ± 17.4	49 ± 21.1	0.004	35 ± 16.2	28 ± 11.9	44 ± 21.1	0.006
APACHE II	16 ± 8.7	15 ± 7.5	17 ± 8.8	NS	15 ± 8.2	13 ± 6.0	18 ± 8.8	NS
SOFA at ICU admission	7 ± 3.5	7 ± 3.9	8 ± 3.8	NS	7 ± 3.8	7 ± 3.7	8 ± 3.5	NS
ICU LOS	8.6 ± 9.1	15.6 ± 20.1	7.0 ± 5.3	0.036	8.8 ± 8.7	12.6 ± 15.8	13.6 ± 19.6	NS
Hospital LOS	24.6 ± 25.9	24.9 ± 28.9	19.2 ± 12.5	NS	23.6 ± 24.9	20.8 ± 16.1	24.9 ± 28.7	NS
ICU mortality (*n* = 10)	4 (10.5%)	4 (10%)	2 (10%)	NS	4 (8.5%)	1 (6.3%)	5 (14.3%)	NS

Values expressed as either % of cases per row or mean ± standard deviation. *p* value of statistical significance

In our database, five patients had a much longer length of stay (51, 53, 60, 68 and 97 days) than the overall group (≤30 days). Four of these five patients presented index values 0.31–2 ((ng/mL)^2^/1000), which may explain this finding

*NS* no statistical significance, *AKI* acute kidney injury, *AKIN* acute kidney injury network definition, *APACHE II* acute physiology and chronic health evaluation II, *ICU* intensive care unit, *LOS* length of stay, *SAPS II* Simplified Acute Physiology Score II, *SOFA* sequential organ failure assessment score

**Fig. 3:**
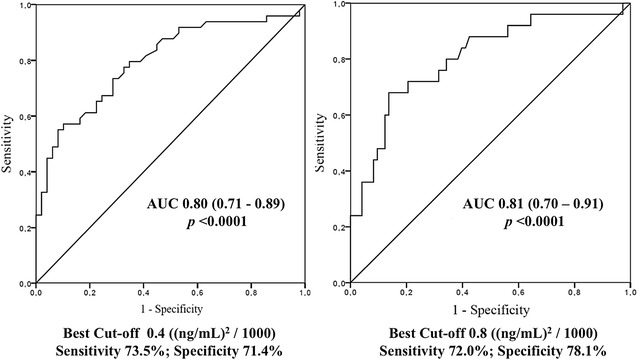
ROC curve for the worst [TIMP-2]·[IGFBP7] index to predict AKI and AKIN ≥ 2. ROC curve and area under the curve (AUC) for the worst [TIMP-2]·[IGFBP7] index concentration within the first 12 h of ICU admission to predict AKI (*left*) and AKIN ≥ 2 (*right*)

We assessed the best predictive cut-off of [TIMP-2]·[IGFBP7] index to predict AKI and AKIN ≥ 2.0 in our population. Table 4 details the sensitivity and specificity of [TIMP-2]·[IGFBP7] test with increasing indexes, as well as the overall accuracy of the test to predict AKI and AKIN ≥ 2. The AUROC curve of the worst value [TIMP-2]·[IGFBP7] within the first 12 h of ICU admission for AKI prediction was 0.798 (0.709–0.886, *p* < 0.0001)) for an index value of 0.40, with sensitivity of 73.5% (95% CI 69.7–77.5%) and specificity of 71.4% (95% CI 67.4–75.4%) (Table 4; Fig. 3). To predict AKIN ≥ 2, the AUROC was 0.805 (0.700–0.909, *p* < 0.0001) with an index value of 0.80, sensitivity of 72.0% (95% CI 68.1–75.9%) and specificity of 78.1% (95% CI 74.6–81.8%).

**Table 4:** Diagnostic and overall accuracy of [TIMP-2]·[IGFBP7] for AKI compared with gold standard AKIN classification

[TIMP-2]·[IGFBP7] cut-off value ((ng/mL)^2^/1000)	Se %	95% CI	Sp %	95% CI	AUC	95% CI
0.3—to predict AKI	81.6	78.2–85.0	59.2	54.9–63.5	0.80	0.71–0.89
0.4—to predict AKI	73.5	69.7–77.5	71.4	67.4–75.4		
0.8—to predict AKIN ≥ 2	72.0	68.1–75.9	78.1	74.6–81.8	0.81	0.70–0.91
2.0—to predict AKIN ≥ 2	48.0	43.6–52.4	90.4	87.8–93.0		

*AUC* area under curve of ROC analysis; *CI* confidence interval; *Se* sensitivity; *Sp* specificity

Patients with index values ≥0.80 (ng/mL)^2^/1000 were older (Table 3). Our threshold did not reveal differences in baseline sCr, baseline eGFR, ICU or hospital length of stay between subgroups. The incidence of shock was higher in patients with index values ≥0.8, although this difference was only marginally significant (*p* = 0.053). However, all patients requiring RRT showed an index value ≥0.8. Thus, compared with the values previously reported in other studies, the observed cut-offs identified 25 additional patients that would not require RRT.

We also evaluated PPV and NPV for both sets of cut-offs (data not shown). However, both sets were equally able to differentiate patients with AKI and AKIN ≥ 2. Although the low incidence of RRT per se increases the NPV, our own specific cut-offs better classified those patients who finally underwent RRT (*p* = 0.007), with a NPV of 100% (95% CI 96.4–100%) for a cut-off >0.8 ((ng/mL)^2^/1000) versus 96.2% (95% CI 92–100%) for a cut-off >2 ((ng/mL)^2^/1000). Values above 2 ((ng/mL)^2^/1000) could only rule out the need of RRT in 40% of cases (95% CI 0–83%).

Taking into account the characteristics of our population (Tables 1, 3), we conducted stepwise logistic regression analysis using as covariates age, sepsis, shock, previous hepatopathy, secondary ARDS, SAPS II, SOFA at admission and the worst value [TIMP-2]·[IGFBP7] for AKI and AKIN ≥ 2 prediction. In our equation, [TIMP-2]·[IGFBP7] index showed an OR of 3.15 (95% IC 1.60–6.17, *p* = 0.001) for AKI prediction and 1.85 (95% IC 1.33–2.57, *p* = 0.001) for AKIN ≥ 2 prediction.

## Discussion

Our results showed that the [TIMP-2]·[IGFBP7] index is a useful predictor of AKI in the first 12 h of ICU admission in both septic and non-septic critically ill patients otherwise free of common AKI risk factors. Our AUROC curves of [TIMP-2]·[IGFBP7] for prediction of both AKI and AKIN ≥ 2 (0.798 and 0.805, respectively) agreed with the results already described in the literature [15, 22–25]. A recently published subgroup analysis of septic patients in sapphire and topaz studies shows similar results [26]. In our study, patients with high index values showed a 3.15- and a 1.85-fold risk to present AKI and AKIN ≥ 2, respectively. The most relevant finding in our study is that a cut-off [TIMP-2]·[IGFBP7] of 0.8 ((ng/mL)^2^/1000) showed a NPV 100% for RRT requirement. Thus, beyond their AKI prognostic capacity, these biomarkers also ruled out the need for RRT below this threshold over the first 12 h of ICU admission.

Sepsis is a common feature in ICU patients [1]. In our study, 40.8% of patients were septic at ICU admission and had a higher incidence of AKIN ≥ 2. Gómez’s unified theory of sepsis-induced AKI may explain why some critically ill patients can present AKI in hyperdynamic and/or non-hypotensive status [27]. AKI and sepsis are reciprocal risk factors. Sepsis triggers inflammation and oxidative stress, and promotes microvascular dysfunction, which can lead to AKI. On the other hand, AKI impairs monocyte cytokine production and elevates plasma cytokine levels [28], acting as a sepsis risk factor. The fact that [TIMP-2]·[IGFBP7] are independently associated with AKI in sepsis is a relevant advantage of these biomarkers compared with more widely described NGAL or cystatin-C [29–31].

Another important finding regards the timing to analyse [TIMP-2]·[IGFBP7]. Repeated determinations of any biomarker in a short period of time may reduce their intrinsic variability and increase their diagnostic or prognostic power. We performed two [TIMP-2]·[IGFBP7] determinations (admission and up to 12 h later) for each patient. In a post-operative cardiac surgery setting, Meersch [23, 32] showed that [TIMP-2]·[IGFBP7] rose as fast as 4 h after surgery (AUROC 0.84, Se 0.92 Sp 0.81) for the highest concentration of [TIMP-2]·[IGFBP7] within the first 24 h. However, in our study, repeated determinations did not improve prediction. This is relevant because a single analysis is easier to implement and decreases costs. The manufacturer recommends repeat testing of patients with an inconclusive result. We would only suggest considering repeating the test in those cases in which clinically their result is difficult to interpret.

Our study tested these biomarkers in a selected ICU population free of common AKI risk factors [33]. Most studies analysing the role of classical or emerging biomarkers in ICU subjects have been done in unselected populations. Accordingly, the number of patients presenting different degrees of renal dysfunction is variable, but usually high. AKI is a common complication of many non-renal hospitalisations [34, 35]; it has been estimated that the incidence of hospital-acquired renal insufficiency is about 7% [36]. Ideally, to describe the characteristics of a new AKI biomarker this incidence should be studied in subjects with a normal baseline renal function. Although our patients may not fully represent an average ICU population in view of our selective inclusion and exclusion criteria, our approach decreases the confounding effect of intra-hospital risk factors to develop AKI, such as exposure to nephrotoxics, hypotension and nosocomial sepsis.

Patients with AKI and/or sepsis showed higher severity scores, and ICU and hospital length of stay, although the latter did not reach statistical difference. Mortality rates remained very low. Thus, we did not find significant differences. This could be partially explained because of our study exclusion criteria. We excluded patients with established anuric AKI at admission, who had the highest risk for poor outcome related to AKI [2, 3], and patients with an expected ICU length of stay <2 days because of their severe condition. Besides, we did not recruit those subjects who had already been hospitalised. In a subanalysis of the PICARD study [37], which analysed the relationship between AKI and sepsis, septic patients either before or after AKI onset presented higher mortality rates than non-septic patients (48, 44 and 21%, respectively).

We also clinically assessed the cut-off values described in the literature. The sapphire and opal studies [15, 16] reported the same cut-off values for the risk of AKI development. In our cohort, the best cut-off index values were 0.4 and 0.8 ((ng/mL)^2^/1000) to differentiate no AKI risk, high risk and very high risk (Table 4). The lower cut-off was very close to the 0.3 previously reported, and it is in keeping with the value cited in the FDA report [38]. Thus, the lower threshold may be helpful to consider before the indication of procedures involving nephrotoxics or to avoid starting RRT in oliguric patients. We also found a consistent NPV above 70% to exclude any degree of AKI. When using our own threshold for very high risk of AKI [0.8 ((ng/mL)^2^/1000)], we did differentiate all patients who finally underwent RRT (*p* = 0.007). The controversy arises in the 0.41–0.8 ((ng/mL)^2^/1000) range; although this subgroup of patients might benefit from a second sampling, this is not supported by our results.

The main limitations in our study are the small sample size and the enrolment in only two centres. Although the overall findings are consistent with recent studies, our thresholds do need further validation. The main strength of the study is the clinical design. The restricted inclusion criteria reduced the size of our cohort and the statistical power, which may also explain the low mortality rate. However, we obtained most baseline sCr and we ensured that patients who developed AKI did not have previous significant CKD. Unlike most observational studies, our proof of model selection of patients also ruled out the added effect of intra-hospital risk factors for AKI.

## Conclusions

Unlike other renal biomarkers, [TIMP-2]·[IGFBP7] predict AKI in both septic and non-septic critically ill patients. These biomarkers are especially useful to promptly differentiate patients without AKI from those with a very high risk of developing AKI. Below the cut-off value of 0.8 ((ng/mL)^2^/1000), the [TIMP-2]·[IGFBP7] index was able to exclude those patients who needed RRT in our study population.

## Additional files

**Additional file 1: Table** **S1.** Main characteristics of study population in subgroups of AKI/non-AKI and septic/non-septic patients. Values expressed as either % per column or mean ± standard deviation; *NS* no statistical significance; *p* value of significance. Abbreviations in alphabetical order. *AKI* acute kidney injury, *AKIN* acute kidney injury definition, *APACHE II* acute physiology and chronic health evaluation II, *ARDS* acute respiratory distress syndrome, *BMI* body mass index, *COPD* chronic obstructive pulmonary disease, *DM* diabetes mellitus, *HTN* hypertension, *RRT* renal replacement technique, *SAPS II* Simplified Acute Physiology Score II, *SOFA* sequential organ failure assessment score. *Exposure to either nephrotoxic or diuretic prior to ICU admission. **Differences in medical admission are due to higher incidence of neurological admissions in subgroup of non-septic patients (24.1% in non-septic subgroup vs. 7.5% in septic patients) and higher incidence of respiratory admissions in septic patients (10.3% in non-septic vs. 47.5% in septic subgroup). As shown in the table, none of the trauma patients included presented concomitant sepsis at ICU admission.

**Additional file 2: Figure** **S1.** Boxplot for [TIMP-2]·[IGFBP7] index values at each determination and depending on AKI and sepsis occurrence. Boxplot represents index values upon admission and up to 12 h later for the overall study population as well as for the subgroups of patients with and without sepsis upon ICU admission. *BM* biomarker, *NS* no statistical significance.

## Abbreviations

TIMP-2: tissue inhibitor of metalloproteinases-2
IGFBP7: insulin-like growth factor-binding protein 7
AKI: acute kidney injury
AKIN: acute kidney injury network definition
ICU: intensive care unit
RRT: renal replacement therapy
sCr: serum creatinine
RIFLE: risk–injury–failure–loss–end-stage renal disease classification
ED: emergency department
CKD: chronic kidney disease
CrCL: creatinine clearance
APACHE II: acute physiology and chronic health evaluation II
SAPS: Simplified Acute Physiology Score
SOFA: sequential organ failure assessment
SIRS: systemic inflammatory response syndrome
Md: median
IQR: interquartile range
PPV: positive predictive value
NPV: negative predictive value
AUROC: area under the receiver operator characteristic curve
OR: odds ratio
Se: sensitivity
Sp: specificity
