# Supplementary material for: Cell-cycle arrest biomarkers in urine to predict acute kidney injury in septic and non-septic critically ill patients
Source: Ann Intensive Care. 2017 Sep 7;7:92. doi: 10.1186/s13613-017-0317-y (PMC5589717; doi:10.1186/s13613-017-0317-y)
Supplement: Supplementary file 1 — Additional file 1: Table S1. Main characteristics of study population in subgroups of AKI/non-AKI and septic/non-septic patients. Values expressed as either % per column or mean ± standard deviation; NS no statistical significance; p value of significance. Abbreviations in alphabetical order. AKI acute kidney injury, AKIN acute kidney injury definition, APACHE II acute physiology and chronic health evaluation II, ARDS acute respiratory distress syndrome, BMI body mass index, COPD chronic obstructive pulmonary disease, DM diabetes mellitus, HTN hypertension, RRT renal replacement technique, SAPS II Simplified Acute Physiology Score II, SOFA sequential organ failure assessment score. *Exposure to either nephrotoxic or diuretic prior to ICU admission. **Differences in medical admission are due to higher incidence of neurological admissions in subgroup of non-septic patients (24.1% in non-septic subgroup vs. 7.5% in septic patients) and higher incidence of respiratory admissions in septic patients (10.3% in non-septic vs. 47.5% in septic subgroup). As shown in the table, none of the trauma patients included presented concomitant sepsis at ICU admission. [file 13613_2017_317_MOESM1_ESM.doc]

**Table S1. Main characteristics of study population in subgroups of AKI/non-AKI and septic/non-septic patients**

Values expressed as either % per column or mean ± standard deviation; NS: no statistical significance; *p*: value of significance.

Abbreviations in alphabetical order:

AKI acute kidney injury; AKIN: Acute kidney injury definition APACHE II: Acute Physiology And Chronic Health Evaluation II; ARDS: Acute respiratory distress syndrome; BMI: Body mass index; COPD: Chronic obstructive pulmonary disease; DM, Diabetes Mellitus; HTN: Hypertension; RRT: Renal replacement technique; SAPS II: Simplified Acute Physiology Score II; SOFA: Sequential Organ Failure Assessment score

* Exposure to either nephrotoxic or diuretic prior to ICU admission.

** Differences in medical admission are due to higher incidence of neurological admissions in subgroup of non-septic patients (24.1% in non-septic subgroup vs. 7.5% in septic patients), and higher incidence of respiratory admissions in septic patients (10.3% in non-septic vs 47.5% in septic subgroup).

As shown in the table, none of the trauma patients included presented concomitant sepsis at ICU admission.

|  | ***Total*** | ***Non AKI*** | ***AKI*** | ***p value*** | ***Non septic*** | ***Septic*** | ***p value*** |
| --- | --- | --- | --- | --- | --- | --- | --- |
| ***n*** | 98 | 49 | 49 | ----- | 58 | 40 | ----- |
| ***HTN (%)*** | 42 (42.9%) | 19(38.8%) | 23 (46.9%) | NS | 23 (39.7%) | 19 (47.5%) | NS |
| ***DM (%)*** | 15 (15.3%) | 5 (10.2%) | 10 (20.4%) | NS | 8 (13.8%) | 7 (17.5%) | NS |
| ***Cardiopathy (%)*** | 18 (18.4%) | 8 (16.3%) | 10 (20.4%) | NS | 10 (17.2%) | 8 (20%) | NS |
| ***COPD (%)*** | 20 (20.4%) | 6 (12.3%) | 14 (28.6%) | NS | 9 (15.5%) | 11 (27.5%) | NS |
| ***Haematologicmalignancy (%)*** | 5 (5.1%) | 4 (8.2%) | 1 (2.0%) | NS | 2 (3.5%) | 3 (7.5%) | NS |
| ***Solid-organ malignancy (%)*** | 8 (8.2 %) | 3 (6.1%) | 5 (10.2%) | NS | 3 (5.2%) | 5 (12.5%) | NS |
| ***Steroids (%)*** | 6 (6.1%) | 4 (8.2%) | 2 (4.1%) | NS | 4 (6.9%) | 2 (5%) | NS |
| ***Hepatopathy (%)*** | 16 (16.3%) | 2 (4.1%) | 14 (23.7%) | 0.002 | 7 (12.1%) | 9 (22.5%) | NS |
| ***Chronic kidney disease*** | 6 (6.1%) | 4 (8.2%) | 2 (4.1%) | NS | 3 (5.2%) | 3 (7.5%) | NS |
| ***Nephrotoxic****^*^* | 17 (17.3%) | 9 (18.4%) | 8 (16.3%) | NS | 7 (12.1%) | 10 (25%) | NS |
| ***Diuretics****^*^* | 16 (16.3%) | 9 (18.4%) | 7 (14.3%) | NS | 7 (12.1%) | 9 (22.5%) | NS |
| ***ICU CHARACTERISTICS*** | | | | | | | |
| ***Medical admission*** | 63 (64.3%) | 30 (61.2%) | 33 (67.3%) | NS | 33 (56.9%) | 30 (75%) | 0.003^**^ |
| ***Surgical admission*** | 29 (29.6%) | 15 (30.6%) | 14 (28.6%) | NS | 20 (34.5%) | 9 (22.5%) | NS |
| ***Trauma admission*** | 6 (6.1%) | 4 (8.2%) | 2 (4.1%) | NS | 6 (10.4%) | 0 (0%) | 0.036 |
| ***Primary ARDS*** | 11 (11.2%) | 5 (10.2%) | 6 (12.3%) | NS | 0 (0%) | 11 (27.5%) | <0.001 |
| ***Secondary ARDS*** | 6 (6.1%) | 0 (0%) | 6 (12.3%) | 0.007 | 3 (5.2%) | 3 (7.5%) | NS |
| ***Mechanical ventilation*** | 79 (80.6%) | 39 (79.6%) | 40 (81.6%) | NS | 44 (81%) | 35 (87.5%) | NS |
| ***Days on mechanical ventilation*** | 7.8 ± 12.9 | 6.9± 9.2 | 8.7± 15.9 | NS | 5.8 ± 9.2 | 10.8 ± 16.7 | NS |
| ***CRP at admission (mg/L)*** | 74.8 ± 93.9 | 56.4 ± 77.3 | 93.2± 105.7 | NS | 39.1 ± 53.2 | 126.9 ± 114.9 | <0.001 |
| ***Lactate at admission (mmol/L)*** | 2.9 ± 3.2 | 2.2± 2.4 | 3.7 ± 3.8 | 0.028 | 2.9 ± 3.7 | 2.9 ±2.3 | NS |
| ***Fluid balance at admission (mL)*** | 507 ± 1357 | 254 ± 812 | 760 ± 1712 | NS | 444 ± 1011 | 598 ± 1751 | NS |
| *Fluid balance at 48h (mL)* | 464 ± 1778 | -169 ± 1106 | -140 ± 1465 | NS | -136 ± 1387 | -181 ± 1158 | NS |
| ***OUTCOME*** | | | | | | | |
| ***SOFA at 24 hours*** | 7.4 ±3.9 | 6.1± 3.5 | 8.7± 4.1 | 0.001 | 6.4 ± 4.1 | 8.9 ± 3.3 | 0.002 |
| ***SOFA at 48 hours*** | 6.8 ±4.3 | 5.5± 3.8 | 8.1± 4.3 | 0.002 | 5.6 ± 4.1 | 8.5 ± 3.9 | 0.001 |
| ***28-day Mortality*** | 12 (12.2%) | 5 (10.2%) | 7 (14.3%) | NS | 7 (12.1%) | 5 (12.5%) | NS |
| ***90-day Mortality*** | 13 (13.3%) | 5 (10.2%) | 8 (16.3%) | NS | 7 (12.1%) | 6 (15%) | NS |
