# Supplementary figures and images for: Cell-cycle arrest biomarkers in urine to predict acute kidney injury in septic and non-septic critically ill patients
Source: Ann Intensive Care. 2017 Sep 7;7:92. doi: 10.1186/s13613-017-0317-y (PMC5589717; doi:10.1186/s13613-017-0317-y)

Figure S1


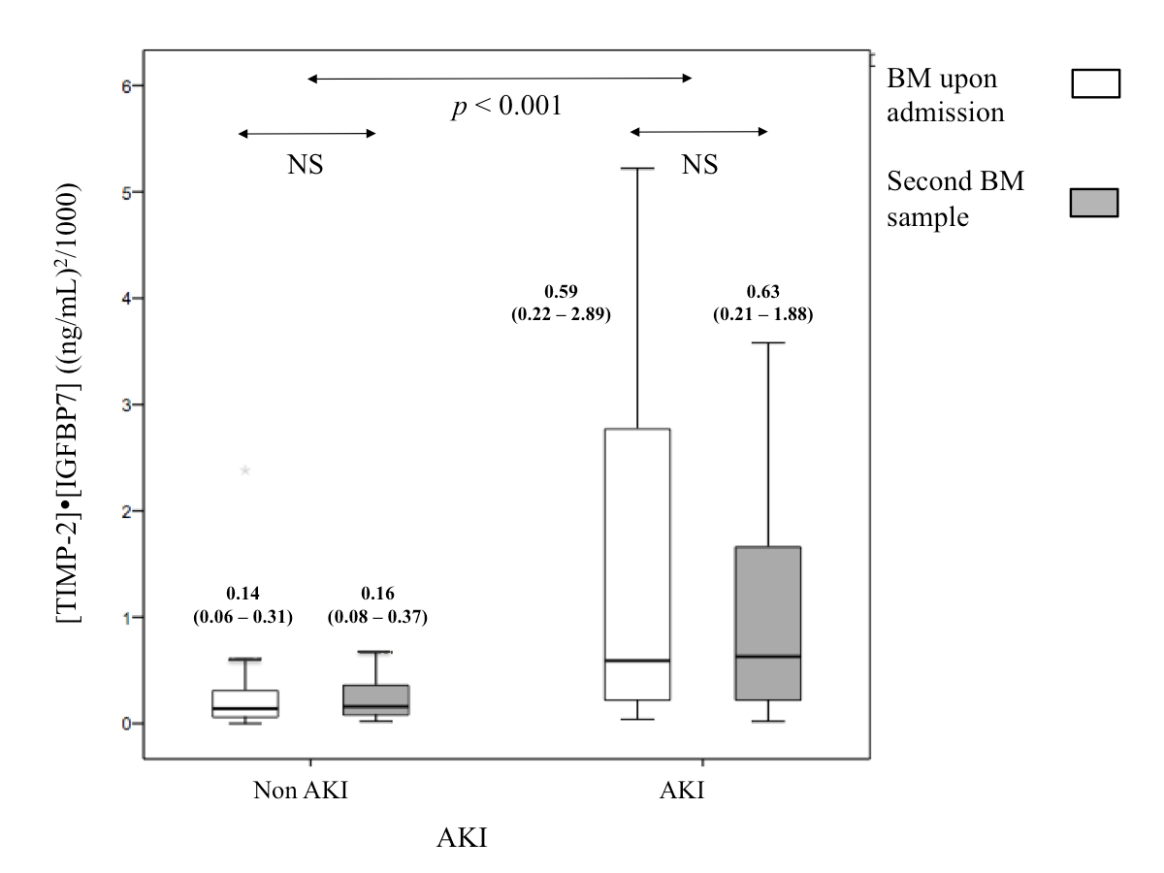

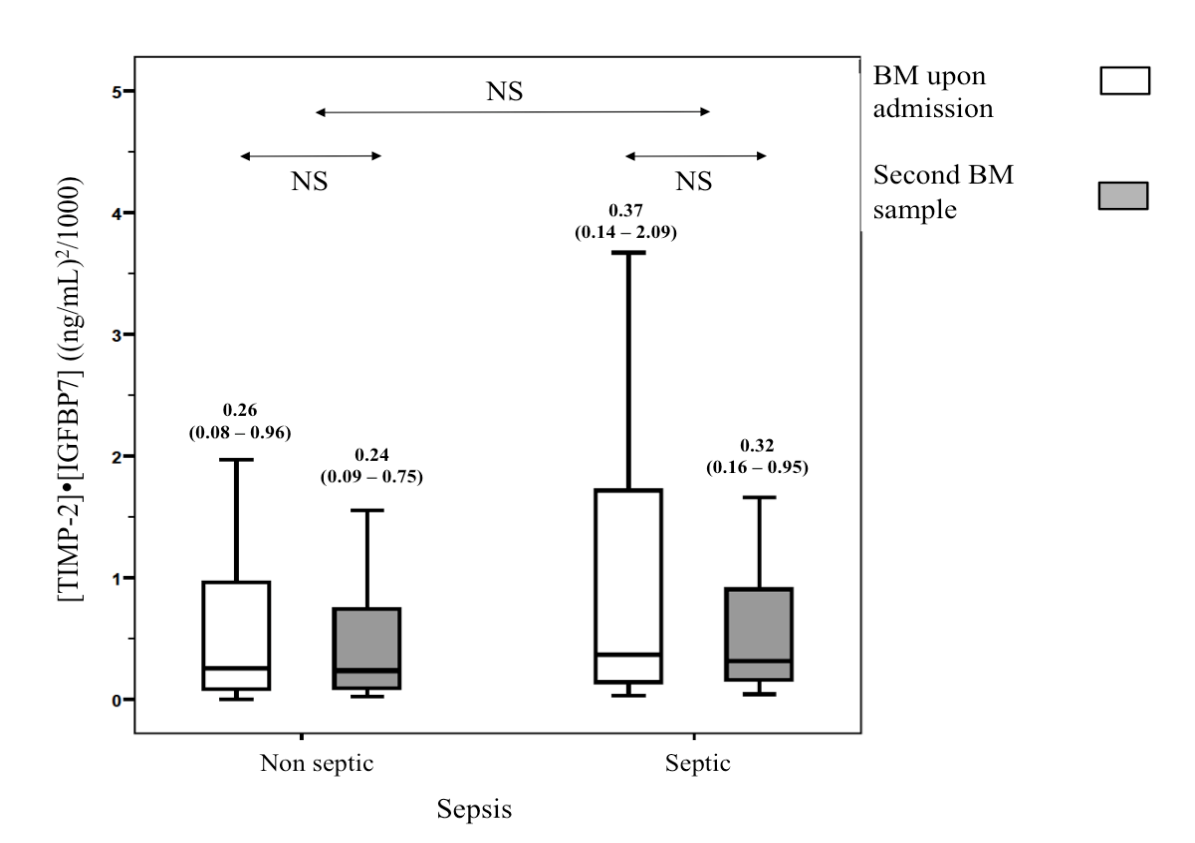

Supplement: Supplementary file 2 — Additional file 2: Figure S1. Boxplot for [TIMP-2]·[IGFBP7] index values at each determination and depending on AKI and sepsis occurrence. Boxplot represents index values upon admission and up to 12 h later for the overall study population as well as for the subgroups of patients with and without sepsis upon ICU admission. BM biomarker, NS no statistical significance. [file 13613_2017_317_MOESM2_ESM.docx]
